# A relationship between body size and the gut microbiome suggests a conservation strategy

**DOI:** 10.1128/spectrum.00294-25

**Published:** 2025-05-21

**Authors:** Tong Xin, Qian Ye, Dini Hu

**Affiliations:** 1 State Key Laboratory of Animal Biodiversity Conservation and Integrated Pest Management, Institute of Zoology, Chinese Academy of Sciences 571195 , Beijing, China; 2 School of Ecology and Nature Conservation, Beijing Forestry University 630065, Beijing, China; 3 Department of Physical Education, Beijing Forestry University 12380, Beijing, China; University of Nebraska-Lincoln, Lincoln, Nebraska, USA

**Keywords:** Felidae, metagenomics, gut microbiota, body size, conservation

## Abstract

A key goal of conservation is to protect the biodiversity of wild species to support their continued evolution and survival. Conservation practice has long been guided by genetic, ecological, and demographic indicators of risk. Cope’s rule suggests that species tend to evolve larger body sizes over time. Here, we provide strong evidence to support the inclusion of body size when formulating wildlife conservation strategies. The gut microbiome can mirror the physiological and environmental adaptation status of the host. This study established a connection between body size and the gut microbiome in the Felidae family using 70 fecal samples collected from 18 individuals through metagenomic data analysis and mining metagenome-assembled genomes (MAGs). Two enterotypes were identified in the Felidae gut: *Bacteroides* and *Clostridium*. Medium-sized felids predominantly harbored *Clostridium*, associated with pathogenicity, whereas large and small felids harbored both beneficial *Bacteroides* and pathogenic *Clostridium*. Species that evolved larger body sizes over time exhibited distinct changes in gut microbial communities, such as enhanced nutrient extraction and metabolic capabilities. Larger felids exhibited a more diverse, stable gut microbiome engaged in metabolic processes and extensive host interactions, indicating an evolved functional role in various biological processes. Conversely, that of smaller felids is less diverse, with more viruses and pathogenic elements primarily involved in chemical synthesis. These findings provide essential insights for developing conservation strategies that consider the nutritional needs of different-sized feline species, control the transmission of pathogens, and allocate resources based on their unique gut microbiome characteristics.

**IMPORTANCE:**

Body size is a fundamental trait that varies greatly among taxa and has important implications for life history and ecology. Cope’s rule suggests that species tend to evolve larger body sizes over time. However, its correlation to body size evolution remains unclear. This study aimed to establish a connection between body size and the gut microbiome in the Felidae family through metagenomic data analysis. Our results support Cope’s rule, illustrating that increased body size correlates with shifts in the gut microbiome, enhancing survival and adaptability.

## INTRODUCTION

A key objective in wildlife conservation is to preserve genetic and phenotypic diversity, ensuring that species can adapt over the long term and survive sustainably in their environments (1). Current strategies for wildlife conservation primarily include enhancing habitat protection, improving laws and regulations, and strengthening international cooperation. In developing these strategies, numerous factors must be taken into account. Presently, international and national conservation frameworks and laws assess threat status by evaluating population trajectories, numbers, range dynamics, and extinction risk (e.g., the IUCN Red List and the Endangered Species Act in the USA). Body size is a fundamental trait that varies greatly among taxa and has important implications for life history and ecology (2–4). Two distinctive processes can lead to changes in average body size within an evolutionary clade. Cope’s rule, a widely accepted pattern, describes the active selection toward increased body size during evolution. Cope’s rule is associated with many benefits, including increased defense against predation, the ability to exploit a greater variety of food sources, and increased survival during periods of environmental stress (5). Cope’s rule has been demonstrated in Canidae (6), Felidae (7), fissiped Arctoidea (8), and oryzomyine rodents (9) but was not supported by Paleogene mammals (10) and Equidae (11). To date, the evolution of mammalian body size has been a focus of ecology, providing important insights into the evolution of mammals and their relationships with the environment (12). Therefore, we advocate that animal size characteristics should be taken into account in the formulation of wildlife protection policies.

The size of animals has a significant impact on their conservation. Different-sized animals play various roles in ecosystems and face distinct challenges. For instance, large animals like African elephants and hippos have advantages in acquiring food and protecting themselves from predators due to their massive size. Smaller animals may rely more on camouflage or rapid escape to avoid their natural enemies (13). Giraffes, with their tall stature, are adapted to the savanna environment and are able to reach leaves that other animals cannot access (14). Besides, larger animals usually have lower reproductive rates, making them more sensitive to environmental changes, and their populations recover slowly. For example, the breeding cycle of the American bison is relatively long, and once threatened, it takes a considerable amount of time for their population numbers to recover (15). Up to now, an increasing number of studies have demonstrated that gut microbiota can serve as an effective indicator of animals’ adaptability to their environment (16). The close symbiotic relationship between gut microbes and their hosts, as well as their interactions with host genetics, can affect the nutrition, immunity, and physiological status of the host (17–19). The gut microbiome is viewed as a complex phylogenetic trait whose variability is shaped and explained through synergistic interactions between host genetic and environmental factors (20). For example, *Firmicutes* and *Bacteroidetes* are the predominant gut microbiota in mammals, and their compositional variations mirror major dietary patterns (21, 22). Within an individual animal species, factors beyond diet also shape inter-individual microbiome variations. Social groups and environmental exposures influence which microbes colonize a host and their abundance (23–25). However, the role of the host’s body size in shaping gut microbial populations has been overlooked by current research. Host genes are intrinsic factors that influence gut microbiome composition. The identification of specific host-controlled microbial communities provides a window into the physiological mechanisms crucial for the adaptation of the host to the environment (26). Our current understanding of how a host’s body size influences the microbiome is limited (26); more studies are needed.

Felidae, a family-level clade of Carnivora, includes all extant cats and several extinct taxa. Almost all living feline species are considered either “endangered” or “threatened” (27). To the best of our knowledge, there is currently no explicit protection strategy in place for these threatened feline species. The exact timing of the origin of Felidae has been debated, but it is widely accepted that Proailurus, which appeared in the latter part of the Oligocene (33.9–23 million years ago), is the most basal taxon (28–30). Extant species within the Felidae family exhibit a wide range of body sizes, ranging from approximately 1 kg for the rusty-spotted cat to approximately 300 kg for the Siberian tiger (31). The wide range of body size in Felidae has inspired research on posture, prey capture, and locomotory ability (32–37). Understanding how body size evolved in this clade and its relationship with the gut microbiome may help us better understand how trait selection occurred and the temporal changes they underwent. To this end, we attempted to ascertain whether body mass evolution in living Felidae could differentiate the gut microbial community, or whether it is better explained by a more complex pattern through the gut microbiota. This information can be used to reconstruct evolutionary history and gain new insights into the development of conservation strategies.

## RESULTS

### Overview of gut metagenome across body size scaling

**Fig 1:**
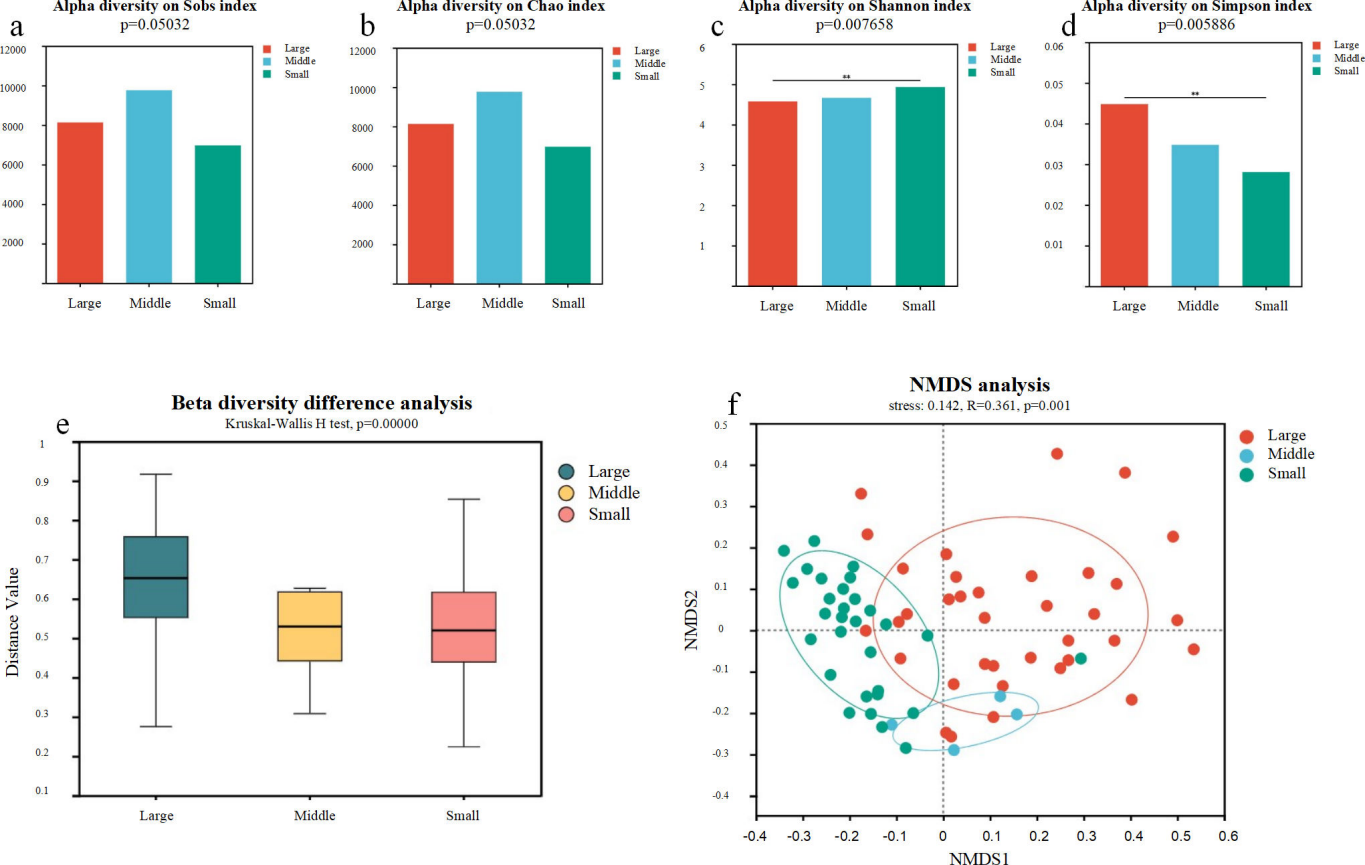
Analysis of alpha diversity differences on Sobs index (a), Chao index (b), Shannon index (c), and Simpson index (d). (e) The Kruskal-Wallis *H* test for the gut microbial community among large, medium, and small felids. (f) The structural differences in the gut microbial community across different body sizes of felids, as revealed by NMDS analysis.

We performed metagenomic shotgun sequencing of 70 fecal samples collected from 18 individuals of the Felidae family, which included 36 samples from large felids, 7 from medium-sized felids, and 27 from small felids. All alpha indices showed significant differences among the three body sizes (Sobs, *P* = 0.050; Chao, *P* = 0.050; Shannon, *P* = 0.008; and Simpson, *P* = 0.006, Fig. 1a through d). Medium-sized felids had the highest microbial richness, followed by large felids and small felids. Small felids displayed higher microbial diversity than large and medium-sized felids. The microbial diversity of medium-sized felids was higher than that of large felids. Beta diversity showed that body size significantly differentiated the microbial community (Fig. 1c, Kruskal-Wallis *H* test, *P* = 0.000). Samples from large, medium-sized, and small felids were separated in the non-metric multidimensional scaling (NMDS) plot (Fig. 1d, ANOSIM, *R* = 0.361, *P* = 0.001).

**Fig 2:**
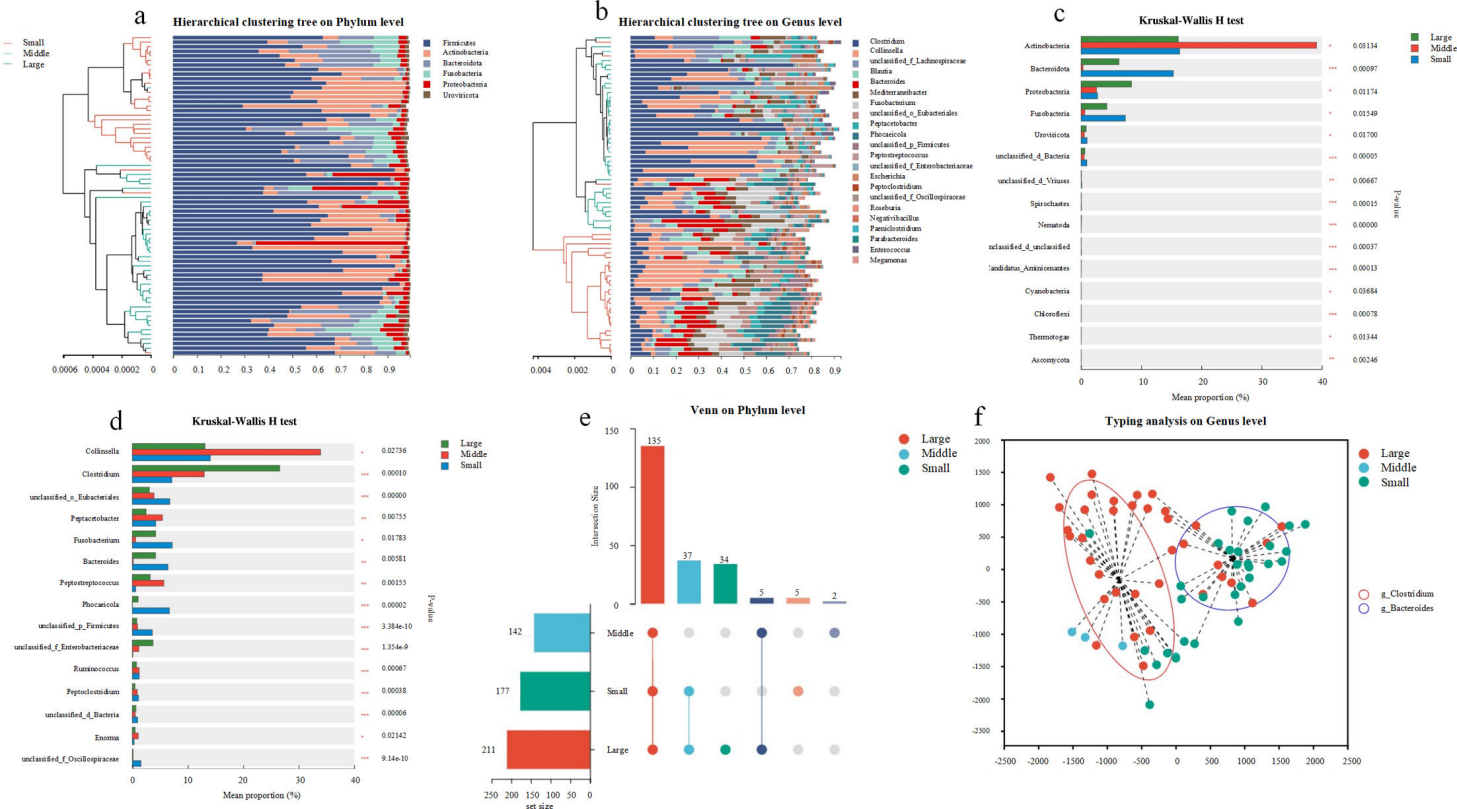
The hierarchical clustering tree illustrates the distribution of gut microbes across various body sizes of felids at both the phylum (a) and genus (b) levels. Significant differences in both phyla (c) and genera (d) were observed among large, medium, and small felids using the Kruskal-Wallis *H* test. The Venn diagram represents the shared and unique types of gut microbes at the phylum level across Felidae of different body sizes (e). (f) The two main enterotypes in the feline gut microbiome.

Next, we identified gut microbial taxa associated with body size. Samples from small felids were clustered separately from those of large and medium felids on the hierarchical tree (Fig. 2a and b). *Firmicutes*, *Actinobacteria*, *Bacterioidota*, *Fusobacteria*, *Proteobacteria*, and *Uroviricota* were the predominant phyla. *Actinobacteria* (*P* = 0.031), *Bacterioidota* (*P* = 0.001), *Proteobacteria* (*P* = 0.012), *Fusobacteria* (*P* = 0.015), and *Uroviricota* (*P* = 0.017) exhibited significant differences in body size (Fig. 2c). The dominant genera consisted of *Clostridium*, *Collinsella*, unclassified_o__*Eubacteriales*, *Peptacetobacter*, and *Fusobacterium* with significant differences among groups (Fig. 2d). We also found that large felids harbored distinct gut microbes, including *Acanthocephala*, *Brachiopoda*, *Bryozoa*, *Foraminifera*, *Hemichordata*, *Microsporidia*, *Negarnaviricota*, *Nemertea*, *Olpidiomycota*, *Porifera*, *Priapulida*, *Rhodophyta,* and *Tardigrada*. In addition, two unclassified phyla were unique to medium-sized felids, while small felids uniquely harbored *Bacillariophyta*, *Cressdnaviricota*, *Kitrinoviricota,* and *Pisuviricota* (Fig. 2e). Based on this obtained data, we suggest that *Bacteroides* and *Clostridium* represent enterotypes in all samples (Fig. 2f). Medium-sized felids exhibited *Clostridium-*associated enterotypes. In contrast, large and small felids exhibited *Bacteroides* and *Clostridium-*associated enterotypes, respectively.

### Functional alternation of gut metagenome across body size scaling

**Fig 3:**
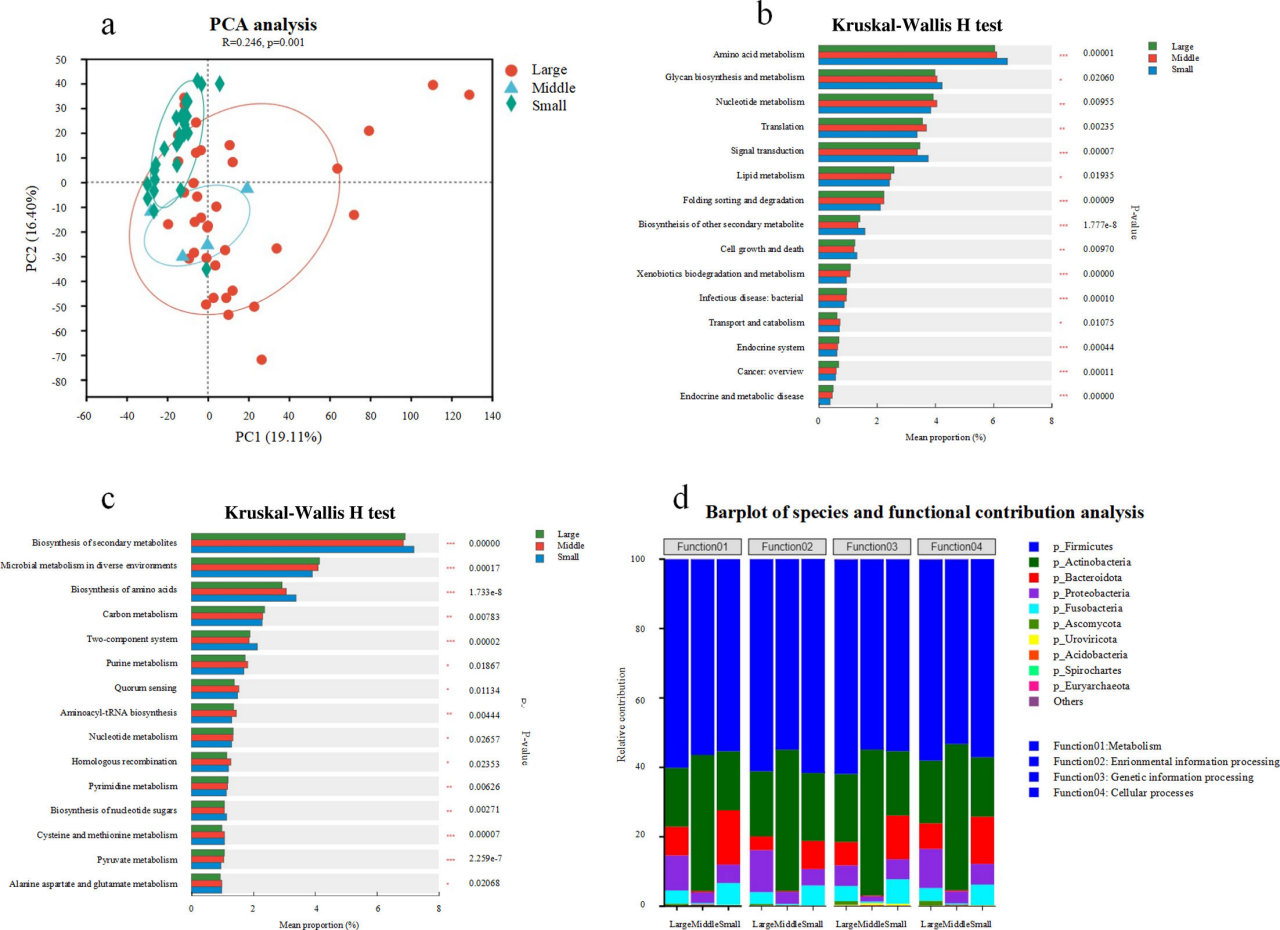
Principal component analysis shows the similarity and differences of the gut microbial community across body size scales (a). Significant differences in Kyoto Encyclopedia of Genes and Genomes Ortholog (b) and KEGG pathway (c) were observed among large, medium, and small felids using the Kruskal-Wallis *H* test. (d) The functional contribution of microbial phyla to Felidae.

Principal component analysis (PCA) revealed a significantly altered functional profile of the gut microbial community across body size scales (Fig. 3a, ANOSIM, *P* = 0.001). In total, 5,215 of the 11,491 Kyoto Encyclopedia of Genes and Genomes (KEGG) Orthologs (KOs) were significantly differentially abundant among groups (*P* < 0.05, Table S1). These KOs corresponded to 218 modules, 382 pathways, and 1,777 enzymes that displayed significant differences among large, medium-sized, and small felids (*P* < 0.05, Tables S2 to S4). Overall, small felids had higher amino acid metabolism (*P* = 0.000) and glycan biosynthesis and metabolism than large and medium-sized felids. Medium-sized felids exhibited the highest nucleotide metabolism, followed by large and small felids (Fig. 3b). At KEGG pathway level 3, large felids showed a higher abundance of microbial metabolism in diverse environments, carbon metabolism, nucleotide metabolism, pyrimidine metabolism, and pyruvate metabolism. In contrast, medium-sized felids were enriched in pathways associated with purine metabolism, quorum sensing, aminoacyl-tRNA biosynthesis, homologous recombination, and alanine, aspartate, and glutamate metabolism (Fig. 3c). Meanwhile, small felids were enriched in the biosynthesis of secondary metabolites, amino acids, two-compound systems, and nucleotide sugars (Fig. 3c). Next, we sought to determine which gut microbial taxa or functions were correlated with body size. Permutational multivariate analysis of variance based on the microbial species level indicated that body size was a stronger factor in changing the gut microbial community composition and structure (*P* = 0.001). Correlation analyses were performed on the microbial community, functions, and related parameters. At the phylum level, *Firmicutes* and *Actinobacteria* in medium-sized felids made a strong contribution to KEGG functions. In contrast, in small and large felids, these two phyla, together with *Bacteroidota* and *Proteobacteria*, have a comparable contribution in large and small felids, similar to those observed in medium-sized felids (Fig. 3d).

### Contribution of gut microbes and their functions to body size scaling

**Fig 4:**
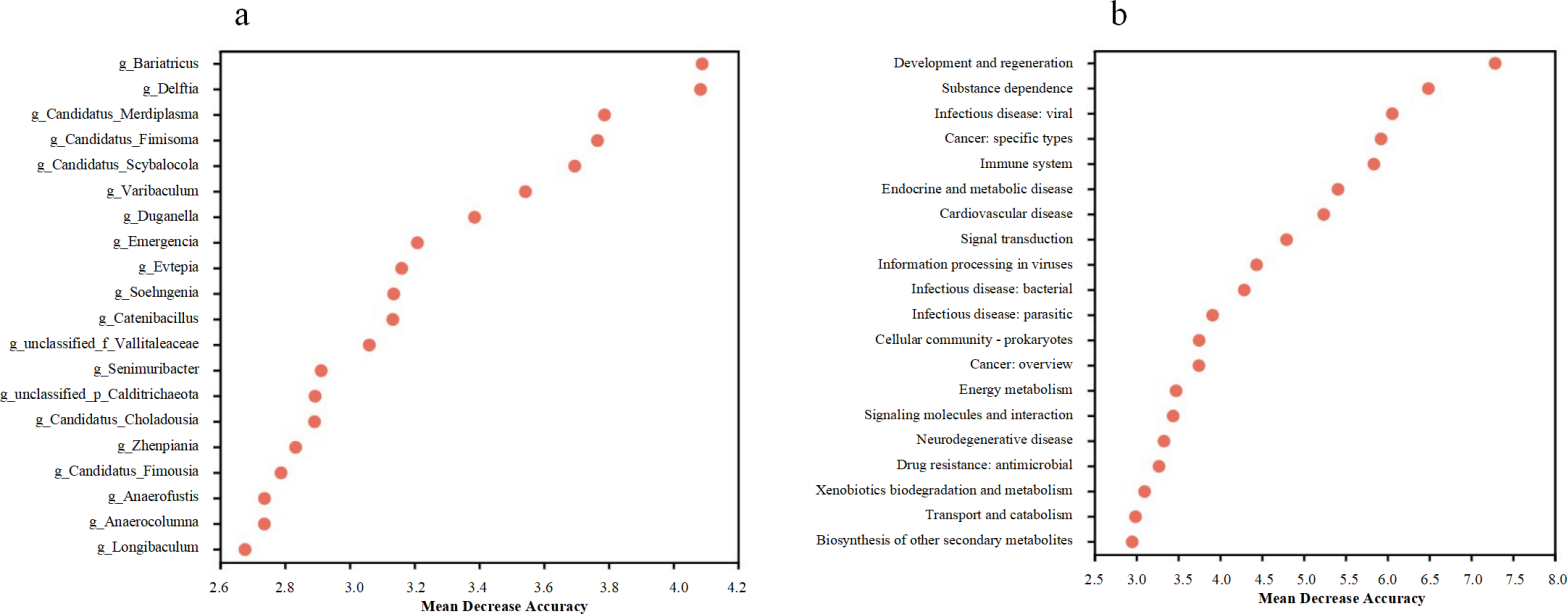
The random forest model detects the microbial genera (a) and their functions (b) in differentiating the body size scales of Felidae.

To investigate whether the gut microbiome can discriminate between mammals with different body sizes, a random forest model at the genus level was constructed to assess its performance. The most important genera were *Bariatricus*, *Delftia,* and *Varibaculum* (Fig. 4a). The key functions associated with these genera included development and regeneration, substance dependence, and viral infectious diseases (Fig. 4b).

### Contribution of microbial species to body size differentiation

**Fig 5:**
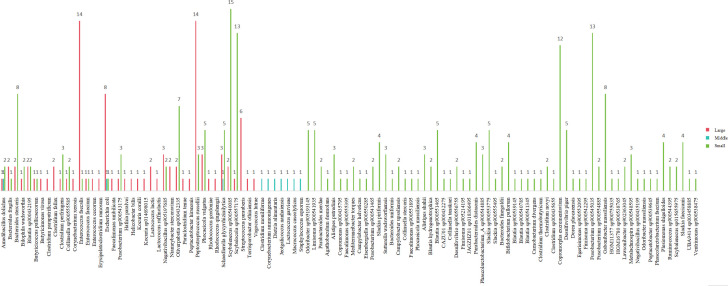
Distribution of high-quality metagenome-assembled genomes and body size variation in Felidae.

We reconstructed a total of 5,700 metagenome-assembled genomes (MAGs) from 70 samples. Of these, 671 MAGs were considered as high quality with completeness ≥ 50% and contamination ≤ 10%. A total of 671 high-quality MAGs were classified into 108 species. Large felids harbored 20 unique species, the most abundant of which were *Streptococcus parauberis*, *Niameybacter stercoravium,* and *Clostridium fallax*. Medium-sized felids harbored seven unique species, including *Clostridium moniliforme*, *Corynebacterium ammoniagenes*, *Dietzia alimentaria*, *Jeotgalicoccus nanhaiensis*, *Lactococcus garvieae*, *Macrococcus caseolyticus*, and *Staphylococcus equorum*. Meanwhile, high abundances of *Odoribacter* sp905193145, *Limisoma* sp900541935, and *Parabacteroides merdae* were observed exclusively in small felids (Fig. 5). Regardless of body size, high-quality MAGs were annotated as adaptive-response sensory kinase SasA, Tyrosine recombinase XerC, and Vitamin B12 import ATP-binding protein BtuD. MAGs in large felids were associated with the production of McpB, MepA, RcsC, and RhaR proteins and glycine reductase, glycosyltransferase, and phosphatase enzymes. MAGs in medium-sized felids were linked to protein Spx and tRNA encoding, while MAGs in small felids were related to the production of DsbD, RcsC, ResA, and RhaR proteins and TonB-dependent receptor, transferase, and Vitamin B12 transporter BtuB (Table 1).

**TABLE 1:** Microbial species with high abundance and their functions

Body size	Bacteria species (the highest completeness)	Protein (number > 5)	Number
Large	*Streptococcus parauberis*	Tyrosine recombinase XerC	9
		Phosphoglycolate phosphatase	5
	*Niameybacter stercoravium*	Multidrug export protein MepA	11
		HTH-type transcriptional activator RhaR	11
		Adaptive-response sensory-kinase SasA	9
		Vitamin B12 import ATP-binding protein BtuD	9
	*Clostridium fallax*	Adaptive-response sensory-kinase SasA	12
		D-inositol-3-phosphate glycosyltransferase	6
		Glycine reductase complex component B subunit gamma	6
		Tyrosine recombinase XerC	6
		Methyl-accepting chemotaxis protein McpB	6
		Sensor histidine kinase RcsC	6
Middle	*Staphylococcus equorum*	Vitamin B12 import ATP-binding protein BtuD	6
		tRNA-Gly(tcc)	5
	*Lactococcus garvieae*	Regulatory protein Spx	13
		Tyrosine recombinase XerC	9
		Vitamin B12 import ATP-binding protein BtuD	5
		tRNA-Met(cat)	5
	*Corynebacterium ammoniagenes*	Fosfomycin resistance protein AbaF	5
		Vitamin B12 import ATP-binding protein BtuD	5
Small	*Limisoma* sp900541935	Tyrosine recombinase XerC	13
		Vitamin B12 transporter BtuB	6
		Undecaprenyl-phosphate 4-deoxy-4-formamido-L-arabinose transferase	5
	*Odoribacter* sp905193145	Thiol-disulfide oxidoreductase ResA	12
		Vitamin B12 import ATP-binding protein BtuD	8
		TonB-dependent receptor P39	7
		Adaptive-response sensory-kinase SasA	7
		Tyrosine recombinase XerC	6
		TonB-dependent receptor P3	6
		Vitamin B12 transporter BtuB	6
		Thiol:disulfide interchange protein DsbD	5
	*Parabacteroides merdae*	TonB-dependent receptor P3	33
		Sensor histidine kinase RcsC	18
		Adaptive-response sensory-kinase SasA	13
		Tyrosine recombinase XerC	10
		Vitamin B12 transporter BtuB	10
		HTH-type transcriptional activator RhaR	10

## DISCUSSION

We explored changes in the gut microbiota of Felidae across variations in body size. Several patterns have emerged. We found a significant correlation between body size and the diversity of the gut microbial community. As body size increased, gut microbial diversity increased while richness decreased. This observation aligns with Cope’s rule (5), which posits that only a few species can adapt and survive amid environmental changes, whereas most species may experience reduced adaptability or extinction. Larger animals possess greater gut capacity, providing more favorable conditions for microorganisms to thrive, resulting in increased gut microbial diversity (38). Furthermore, the patterns of gut microbes were significantly associated with body size, which supports the conclusion that body size is a crucial driver of variation in the gut microbial community (38, 39). We found that small felids carry unique viruses, such as *Cressdnaviricota* (40), *Kitrinoviricota* (41), and *Pisuviricota* (42). However, these specific viruses tend to diminish with increasing body size, being replaced by various planktonic organisms and parasites (43–53). This transition could be attributed to larger animals having a more diverse diet. As stated in Cope’s rule (5), increased body size enables access to a wider range of food resources for survival.

In addition, community-level analysis suggested that microbial function was significantly related to body size. These results suggest that the gut microbiota in small felids are mainly involved in chemical synthesis, including the biosynthesis of secondary metabolites, amino acids, and nucleotide sugars. Meanwhile, the gut microbiota of large and medium-sized felids mainly participates in microbial metabolic processes. These processes include the metabolism of carbon, glutamate, nucleotide, purine, pyrimidine, and pyruvate. Gut microbial metabolism plays a critical role in the health and disease resistance of the host (54). This suggests that larger body sizes often improve an animal’s ability to maintain metabolism, raise thermal inertia, accommodate climatic variation, and withstand starvation (55, 56). Chemical synthesis by gut microorganisms refers to the process through which microorganisms use nutrients, such as carbohydrates, fats, and proteins, in their environment to synthesize organic compounds via metabolic pathways (57). This requires small animals to obtain more food to support the chemical synthesis of their gut microbiota. However, under conditions of limited activity range and food shortage, their risk of extinction increases (56).

The gut microbiota can be classified into several enterotypes, which are typically stable but can be influenced by the diet and health of the host (58). We identified two enterotypes in the guts of Felidae: *Bacteroides* and *Clostridium. Clostridium* was prevalent in medium-sized felids. However, large and small felids were associated with *Bacteroides* and *Clostridium. Bacteroides* species are regarded as beneficial organisms, gut competitors, and opportunistic pathogens (59). *Clostridium* species are associated with diseases in animals (60, 61). The enterotypes of large and small felids were a combination of beneficial and pathogenic bacteria, whereas the enterotype of medium-sized felids was dominated by pathogenic bacteria. The evolution of animals is a process of adaptation to abiotic environments (62). Environmental changes have led to an increase in the geographic range of pathogenic species and their vectors (63). Medium-sized felids serve as a transitional group, acquiring more pathogenic bacteria alongside beneficial species, indicating that evolution is underway. Functional analysis revealed that four phyla perform important functions in the survival of large and small felids, but only two phyla perform important functions in their medium-sized counterparts. Thus, medium-sized felids are less stable from the perspective of the gut microbiome.

More importantly, we found that the body size was significantly associated with the abundance of *Bariatricus*, *Delftia,* and *Varibaculum*. The distinct functions are development and regeneration, substance dependence, and viral infectious diseases. *Bariatricus*, *Delftia*, and *Varibaculum* are bacterial genera found in various environments, including animal guts. *Bariatricus* can potentially influence the metabolic processes in the gut (64). Members of *Delftia* are more frequently encountered in the environment than in the gut. Given their ability to degrade complex organic pollutants, it is possible that they could contribute to the breakdown of similar compounds in the gut (65). Species of *Varibaculum* are known for their ability to aid digestion and nutrient absorption (66). It is worth noting that the body sizes of these genera can vary greatly depending on factors such as diet and health status, which aligns with Cope’s rule. These findings support the notion that the progression of evolution is dependent on continuous adaptation, including adaptation to diet, environment, and resources.

Finally, we constructed MAGs from Felidae to elucidate the bacterial functions underlying changes in body size. Unique and highly abundant bacterial species in large felids, such as *Streptococcus parauberis*, *Niameybacter stercoravium*, and *Clostridium fallax*, produce proteins and enzymes with significant biological functions, including McpB, MepA, RcsC, RhaR, glycine reductase, glycosyltransferase, and phosphatase. McpB plays a key role in maintaining the balance of the immune system (67). MepA maintains the material balance inside cells by recognizing and transferring various molecules, and its expression is closely related to bacterial drug resistance (68). RcsC and RhaR play important roles in the bacterial stress response, virulence factors, and bacterial metabolism (69, 70). Glycine reductase is involved in key steps in the amino acid metabolic pathway in bacteria (71). Glycosyltransferases participate in glycosyl transfer in various organisms (72). Phosphatases are involved in biological processes such as energy metabolism, apoptosis, and DNA repair (73). *Staphylococcus equorum*, *Lactococcus garvieae,* and *Corynebacterium ammoniagenes* are prevalent in the gut of medium-sized felids and are known for their ability to produce proteins such as Spx and tRNA molecules. Spx serves as a crucial regulatory protein that plays multiple roles in bacterial nitrogen and phosphorus metabolism, environmental adaptation, and pathogenic processes (74). It interacts with other proteins to respond to intracellular and extracellular levels of nitrogen and phosphorus, thereby regulating the expression of corresponding genes and influencing the physiological state and pathogenicity of the bacteria. Small felids are populated by bacteria such as *Limisoma* sp900541935, *Odoribacter* sp905193145, and *Parabacteroides merdae*, which produce DsbD, RcsC, ResA, RhaR, TonB-dependent receptor, transferase, and the vitamin B12 transporter BtuB. DsbD is responsible for activating or maintaining the structure of virulence factors in certain pathogenic bacteria, allowing them to survive within the host and cause disease (75). RcsC, a protein involved in regulating cell wall synthesis and stress responses in bacteria, plays a key role in bacterial growth and adaptation to environmental changes (76). ResA is a crucial protein involved in malarial infection (77). RhaR participates in regulating the expression of genes related to sugar metabolism and stress responses under environmental conditions, significantly affecting bacterial survival and reproduction (70). TonB-dependent receptors play an important role in bacterial nutrient acquisition (78). Transferases are vital for numerous biochemical processes, such as metabolic pathways, DNA replication and repair, and protein modification (79). The vitamin B12 transporter BtuB is a bacterial protein responsible for transporting vitamin B12 (80). The results of the MAG analysis enhance our knowledge of how the gut microbiome supports Cope’s rule. The gut microbiota of small animals only affects the survival, reproduction, and disease occurrence of bacteria. As evolution continues, the gut microbiota of larger animals participates beyond bacterial metabolism and is involved in different biological processes and molecular mechanisms, maintaining normal cell function and vital life activities.

In conclusion, the metagenomic analysis of Felidae provides new insights into understanding Cope’s rule. The gut microbiome of the small felids is vibrant and is considered to be at an early stage of evolution. It is characterized by low diversity and a high prevalence of viruses and pathogenic elements. The primary function of this microbiome is chemical synthesis, which supports bacterial growth and reproduction. In contrast, the gut microbiome in large felids is diverse and approaches stability. These microbes participate in metabolic processes, interact with the host, and are involved in a wide range of biological processes. The study’s revelation that small and large feline species exhibit significant differences in their gut microbiome composition and function offers crucial insights for the development of feline conservation strategies. For instance, when designating habitats, it is imperative to consider the distinct nutritional requirements of felids based on their size, manage the transmission of pathogens effectively, and allocate resources according to the unique characteristics of their gut microbiomes.

## MATERIALS AND METHODS

### Metagenomic data collection

Fecal samples of felids were collected between April and June 2023 from the Beijing Wildlife Zoo, Beijing Zoo, Breeding Center of Beijing Zoo, Chongqing Zoo, and Xining Wildlife Zoo. All fecal samples were immediately frozen in dry ice after collection and transported to the laboratory for storage at −80°C until further processing. Detailed information on sample collection can be found in Hu et al. (81). In accordance with body weight and body length, the sampled felid species are classified into three categories: small felids (those weighing less than 20 kg and body length less than 100 cm), medium-sized felids (those weighing between 20 and 40 kg and body length over 100 cm), and large felids (those weighing over 40 kg and body length over 100 cm) (Table 2).

**TABLE 2:** The detailed information of the sampled Felidae, including species name, sample size, body size, body weight, body length, and collection site^*a*^

Feline species	Sample size	Body size	Body weight (kg)	Body length (cm)	Collection site
*Prionailurus bengalensis*	11	Small	1.5–5	36–66	Breeding Center of Beijing Zoo
	1			36–66	Chongqing Zoo
*Felis bieti*	5		5	60–80	Xining Wildlife Zoo
*Otocolobus manul*	5		5	60	Xining Wildlife Zoo
*Pardofelis temminckii*	1		12–16	75–100	Chongqing Zoo
*Caracal caracal*	1		13–18	66–76	Beijing Zoo
*Leptailurus serval*	1		13.5	85	Breeding Center of Beijing Zoo
	2			85	Beijing Zoo
*Neofelis nebulosa*	2	Middle	20	75–110	Chongqing Zoo
*Acinonyx jubatus*	1		30–40	112–135	Beijing Zoo
*Lynx lynx*	1		30	130	Xining Wildlife Zoo
	3			130	Beijing Zoo
*Panthera uncia*	9	Large	40–60	110–130	Xining wildlife park
*Panthera pardus*	1		50–100	100–150	Beijing Zoo
*Panthera pardus* (black)	1		50–100	100–150	Chongqing Zoo
*Panthera onca*	1		80–180	112–185	Beijing Wildlife Zoo
*Panthera leo*	1		110–160	250–320	Chongqing Zoo
	5			250–320	Beijing Zoo
*Panthera leo* (white)	1		110–160	250–320	Beijing Wildlife Zoo
	1			250–320	Beijing Zoo
*Panthera tigris* ssp. *Tigris* (white)	6		135–230	200–290	Chongqing Zoo
	3			200–290	Beijing Zoo
*Panthera tigris* ssp. *Amoyensis*	3		150	250	Chongqing Zoo
*Panthera tigris* ssp. *Altaica*	2		170	200–290	Beijing Wildlife Zoo
	2			200–290	Beijing Zoo

^*a*^ Body weight refers to the total mass of an animal’s body. Body length refers to the linear measurement of an animal’s body from its head to the end of the tail. The exact number or range for body weight and body length is shown.

### Bioinformatic analysis

The methodology outlined by Hu et al. (81) was followed. The E.Z.N.A. Soil DNA Kit (Omega Bio-tek, U.S.) was used to extract total DNA from the collected feces, according to the manufacturer’s protocol. TBS-380, NanoDrop 2000, and 1% agarose gels were used to assess DNA concentration, purity, and integrity, respectively. The DNA extract was fragmented to an average size of approximately 400 bp using a Covaris M220 (Gene Company Limited, China) for paired-end library construction. NEXTFLEX Rapid DNA-Seq (BioScientific, USA) was used to construct a DNA library. Adapters containing the full complement of the sequencing primer hybridization sites were ligated into the blunt ends of the fragments. Qualified DNA libraries following PCR amplification were sequenced on an Illumina NovaSeq platform (Illumina Inc., San Diego, CA, USA). Trimmomatic and Fast QC were used for the quality control of all metagenomic sequences. Bowtie2 was used to remove the host information, after which Megahit was employed to assemble reads. Kraken was used to classify the microbes. MetaBAT2 was used for the genome binning. The redundant bins were removed using the dRep software. CheckM was used to determine the final quality of the bins, including completeness and contamination. The tRNA and rRNA genes of MAGs were predicted using Barrnap (https://github.com/tseemann/barrnap) and tRNAscan-SE, respectively. Phylogenetic analysis of the MAGs was conducted using the GTDB. Functional annotation was performed using the KEGG database (https://www.kegg.jp). Protein-coding genes and protein products were predicted using Prokka software (https://github.com/topics/prokka).

### Statistical analysis

NMDS, ANOSIM, Kruskal-Wallis *H* test, Venn, PCA, hierarchical tree, and random forest models were performed using R 4.3.0.

## Data Availability

The raw sequence data reported in this paper have been deposited in the Genome Sequence Archive (Genomics, Proteomics & Bioinformatics 2021) in the National Genomics Data Center (Nucleic Acids Res 2022), China National Center for Bioinformation/Beijing Institute of Genomics, Chinese Academy of Sciences, under accession number CRA014688.

## SUPPLEMENTAL MATERIAL

Table S1 spectrum.00294-25-s0001.xlsx Distribution of KEGG Orthologs (KOs) with significant differences across different groups.

Table S2 spectrum.00294-25-s0002.xlsx Distribution of significantly changed modules related to KOs across different groups.

Table S3 spectrum.00294-25-s0003.xlsx Distribution of significantly changed pathways related to KOs across different groups.

Table S4 spectrum.00294-25-s0004.xlsx Distribution of significantly changed enzymes related to KOs across different groups.

ASM does not own the copyrights to Supplemental Material that may be linked to, or accessed through, an article. The authors have granted ASM a non-exclusive, world-wide license to publish the Supplemental Material files. Please contact the corresponding author directly for reuse.
